# Editorial

**DOI:** 10.1120/jacmp.v4i1.2536

**Published:** 2003-01-01

## Editorial

The online Journal of Applied Clinical Medical Physics (JACMP) has moved editorial operations to Rochester, Minnesota. I am being ably assisted by Sarah D. Evers, who will become known to many of you as “Manuscript Manager.” Four issues are planned for 2003, which is Volume 4, with this Winter issue being the first. A 10‐paper minimum per‐issue goal was set, and this was exceeded. The “pipeline” of articles under consideration is staying steady at about 20. We are discussing with AIP their service to put accepted manuscripts online the instant a final copy is submitted so an issue will “build.” This could cut availability time down by up to three months and certainly adds value to potential authors.

I would be very remiss if I did not specifically acknowledge the work of Peter Almond (Editor‐in‐Chief for Volumes 1‐3), as well as Georgeanne Moore (Manuscript Manager for Volumes 1‐3). As I am finding out, there is a ton of little details that have to be attended to. The fact that the Journal has grown (about 60% per year) and thrived under their “watch” is a testimony to their dedication to the task. The ACMP and the worldwide medical physics community owe them a debt of gratitude that really cannot be repaid adequately.

It became painfully obvious after taking over the helm that additional Associate Editors (AE) were needed. Four new Associate Editors have been added. In the area of Therapy Physics we have added John Gibbons, Tim Solberg, and Gino Fallone. We are also extremely pleased that Walter Huda has agreed to join the Diagnostic Imaging AE “team.” Editorial Board members and reviewers are to be thanked profusely for volunteering their expertise to insure a consistent and high‐ quality product. I have been very pleased to see the effort that goes into the AE and their reviewer's deliberations. This is a truly peer‐reviewed journal.

JACMP is available free to a worldwide audience, and is now an official publication of the International Organization of Medical Physics (IOMP). As such, the Editorial Board is developing strategies to increase international submissions and online access numbers. Informal discussions are underway to see whether there could be a role for JACMP in placing online nonjournal published Tech‐ nical Reports from some major medical physics societies, increasing worldwide availability and helping carry out their international scientific mission(s).

As I jot down these “musings,” I am heading to an Online Electronic Journal Publishing Meeting at AIP in College Park. It will be interesting to learn of others' experiences. I am particularly interested in being updated on the changes to the electronic submission system. The current one is quirky and had certain features I find suboptimal (like the authors get to preselect the Associate Editor!).

The principal goal of the Editorial Board (below) remains that JACMP be the first choice source of rapidly published and thoroughly peer‐reviewed information for immediately useful clinical medical physics information. JACMP is seeking high‐ quality papers that deal with the application of the observations, data, and/or procedures in the clinic. Class examples include technology, information systems, treatment techniques, quality assurance, and procedure guidelines. Immediate usefulness in the clinic should be the hallmark of a JACMP sub‐ mission. Please consider JACMP for some of your future manuscripts. We promise fair and speedy review as well as the ability to reach a wide international audience.

I look forward to visiting you next time in the “Editorial.” Please feel free to email me (ecmc@mayo.edu) with any suggestions for how the Journal can better serve your needs as a reader or, better still, an author!!

Edwin C. McCullough

Editor‐in‐Chief

